# Applications of Plant Biotechnology: In Vitro Propagation and Plant Transformations

**DOI:** 10.3390/plants15192892

**Published:** 2026-09-22

**Authors:** Humberto Prieto

**Affiliations:** Biotechnology Laboratory, Institute of Agricultural Research–La Platina, Santiago 8831314, Chile; hprieto@inia.cl

## 1. Introduction

Modern plant biology stands at a transformative juncture where integrating advanced tissue culture, high-throughput genomics, and precise genome editing is driving a new era of agricultural resilience. Methods are needed to speed up the breeding of crop varieties with improved agronomic and physiological traits to address the urgent requirement for global food security under increasing climate variability. This Special Issue, “Applications of Plant Biotechnology: In Vitro Propagation and Plant Transformations”, contains twelve papers that bridge basic cellular biology with the use of commercial crops. This collection demonstrates how optimized regeneration systems, refined transformation pipelines, and emerging molecular boosters converge to overcome long-standing issues in plant biotechnology. These studies offer an innovation pipeline for overcoming the persistent challenges with tissue culture, transformation efficiencies, gene expression limits, and accurate genomic editing, which will impact 21st century plant breeding.

### 1.1. Cell Fate and Ploidy Manipulation in In Vitro Systems

The establishment of any successful genetic transformation or editing platform begins with a deep physiological understanding of cellular pluripotency and regeneration. In woody perennials, in vitro multiplication is frequently limited by apical dominance and culture heterogeneity, both of which are regulated by complex hormone dynamics. To address these limitations, Grira et al. [1] investigated the influence of donor material (topophysis) position and culture density on the micropropagation of wild olive (*Olea europaea* subsp. *sylvestris*). The highest propagation was obtained with middle-section explants grown at a density of 30 explants per vessel, with up to 3.5 shoots and 17.3 nodes per plant. This dense microenvironment induces a local stress-like response and alters endogenous cytokinin migration. Active aromatic free bases accumulate in shoot apices (up to 1476 pmol/g DW), whereas isoprenoid conjugates migrate to the base. This solves the apical dominance problems in forestry. The study exemplifies how manipulating endogenous cytokinin gradients can systematically resolve apical dominance, an issue covered in several contributions in this Special Issue.

In addition to tissue multiplication, in vitro cultures are useful tools for chromosome doubling, which can improve desirable agronomic traits as well as increase the levels of secondary metabolites and stress resilience. Tang et al. [2] developed a highly efficient polyploidy induction system for Longshan *Lilium lancifolium*, a valuable medicinal and edible local plant in China. The stable, homozygous tetraploid line ‘JD-12’ (68 chromosomes) was obtained by treating regenerated shoots with 0.10% colchicine for 48 h. This line displayed high phenotypic gigantism with bigger cells and thicker leaves, lower stomatal density (9.66 mm^−2^ versus 29.75 mm^−2^), and evident genetic variation (6.10% using ISSR markers). JD-12 offers a robust cellular basis for improving lily breeding initiatives. The study reinforces the importance of controlling mitotic disruption as a reproducible strategy to generate elite germplasm, complementing the regeneration-focused studies also included in this Special Issue.

For woody crops with long juvenile periods, direct organogenesis from somatic tissues represents a rapid, clonal pathway that minimizes somaclonal variation. Maletich et al. [3] studied the direct organogenesis of 22 genotypes of *Vitis vinifera* and developed an efficient *Agrobacterium*-mediated transformation system for the elite cultivar ‘Podarok Magaracha’. This system overcomes the bottleneck of somatic embryogenesis with a finely tuned three-stage cultivation cycle (dark induction, light multiplication, and dark elongation) over 11 weeks, with a balance of 6-benzyladenine (BA) and indole-3-butyric acid (IBA). The approach allowed for stable transformation (2.0% efficiency), with single or double copies of *gfp* and *nptII* cassettes, validating this protocol as a scalable, genotype-compatible organogenesis pipeline for viticulture biotechnology. The contribution highlights how genotype-tailored organogenic routes can be standardized into scalable transformation platforms, a recurring objective of multiple studies in this Special Issue.

### 1.2. Optimizing Delivery: Transformation Systems and Translational Boosters

For many nonmodel or ecologically significant plants, the lack of stable transformation protocols has delayed functional genomic characterization. Common purslane (*Portulaca oleracea* L.) is an annual succulent herb serving as a key model for studying C4/CAM photosynthetic plasticity. Xu et al. [4] established an effective, leaf-derived somatic-cell regeneration and *A. tumefaciens*-mediated transformation protocol for common purslane. They systematically evaluated a combination of hormones on 27 different accessions and found that somatic cells from leaf tissues have stronger dedifferentiation capacities. The shoot regeneration was more than 60% on MS medium supplemented with 2 mg/L 6-BA in 22 genotypes. They used a threshold of 50 mg/L kanamycin for selection (hygromycin was highly inhibitory) and recovered 17 T0 transgenic lines with stable Mendelian inheritance in the T1 progeny. The study demonstrates that systematic hormone screening and selection optimization can transform previously recalcitrant species, similar to the breakthroughs reported here for monocots and legumes.

In monocot species, establishing transient platforms is crucial for bypassing long regeneration cycles and rapidly validating gene functions. Wang et al. [5] developed two quick transient expression systems for Welsh onion (*Allium fistulosum* L.), an important pungent vegetable with a complex “super-genome” worldwide. By optimizing *Agrobacterium rhizogenes* Ar. Qual stem-disc infection parameters (OD_600_ = 0.3, 100 µM acetosyringone), they achieved an 88.75% hairy root induction efficiency. They also optimized the leaf-derived protoplast isolation protocol through cellulase and macerozyme digestion, which generated 3.3 × 10^6^ viable protoplasts g^−1^ of fresh weight with 90% viability. The GFP-reporter plasmid was transfected into protoplasts via PEG, and transient expression efficiency was confirmed at 10.47%, providing a molecular toolkit for studying subcellular localization and validating gene editors in *Allium* crops. The dual-platform approach offers a versatile toolkit for functional genomics and is representative of the general trend in this Special Issue of combining transient and stable systems for complementary molecular validation.

Transgene overexpression at high transcriptional levels upon delivery may induce post-transcriptional gene silencing or cellular toxicity. To overcome this issue, Guo et al. [6] designed an artificial sequence element, complementary to the internal *18S rRNA* of the 40S ribosomal subunit, named active ribosomal RNA complementary (ARC). They found that six tandem repeats of ARC (6 × ARC) increased reporter gene translation up to 130-fold without affecting the mRNA transcript levels in transient and stable transformation assays in *Chirita pumila* and *Arabidopsis thaliana*. The integration of 6 × ARC with tissue-specific promoters resulted in the extreme and localized amplification of transgenic phenotypes, such as the repression of stamen development in *C. pumila* or the induction of intense anthocyanin accumulation in *Arabidopsis* pedicels. Their results suggest that 6 × ARC is a powerful and highly conserved tool for fully manifesting exogenous gene functions. This translational booster represents a unifying conceptual advance in this Special Issue, demonstrating how synthetic regulatory elements enhance expression independent of promoter strength or transformation method.

### 1.3. Single-Cell CRISPR/Cas9 Platforms for Precision Breeding

The advent of CRISPR/Cas9 has revolutionized crop breeding from classical transgenesis to precise genome editing. However, editing multicellular explants often leads to chimerism, with only a subset of the regenerated tissues containing the desired genetic modification. Researchers are increasingly integrating CRISPR/Cas9 delivery with single-cell protoplast and somatic embryogenesis systems to produce uniformly edited, nonchimeric lines. Using CRISPR/Cas9 editing, Potsenkovskaia et al. [7] investigated the functional role of the *LEC1* ortholog *MtNF-YB10* in the model legume *Medicago truncatula*. Targeting the conserved B domain with a guide RNA driven by the U6-26 promoter, they recovered homozygous loss-of-function mutants with a 7.3% editing efficiency. In the *mtnf-yb10* mutants, zygotic embryogenesis seemed normal, but seed germination percentages and root length were considerably lower and the ability for in vitro somatic embryogenesis was completely abolished in the mutants, providing unequivocal genetic evidence of the essential function of the gene in the maintenance of embryogenic competence. The study underscores the necessity of single-cell-based regeneration systems for achieving uniform edits, a principle further expanded by the rice-focused study in this Special Issue.

To address the issue of chimerism in polyploid monocots, Barrera et al. [8] created a reproducible protocol for protoplast isolation and regeneration in two elite Chilean temperate japonica rice cultivars, ‘Ónix’ (black-grained) and ‘Platino’ (short-wide-grained). They isolated viable protoplasts (70–99% viability after treatment with 1.5% cellulase and 0.75% macerozyme in 0.6 M AA medium) from seed-derived embryogenic calli. Plantlets were regenerated via encapsulation in calcium alginate beads cocultured with nurse cells. The average number of regenerated seedlings per gram of callus was 12 for ‘Platino’ and 20 for ‘Ónix’. Transient PEG-mediated transfection of a CRISPR/Cas9 plasmid targeting the *DROUGHT AND SALT TOLERANCE* (*OsDST*) gene successfully edited the locus, validating this protoplast-based pipeline as a highly scalable and genotype-compatible engine for nontransgenic, DNA-free editing in cold-adapted rice germplasm. This pipeline shows how genotype-adapted editing systems can be incorporated into breeding programs and reflects this Special Issue’s themes of precision and scalability.

### 1.4. Physiological Trade-Offs: New Biotherapies and Regulatory Gates

As sophisticated genetic modifications transition from the laboratory to the field, the physiological trade-offs between defense pathways and crop productivity must be understood. Miroshnichenko et al. [9] studied the effect of the constitutive overexpression of jasmonate biosynthesis genes (*AtAOS*, *TaAOS* and *AtOPR3*) in emmer and bread wheat. Although the jasmonate levels of the transgenic lines were elevated under stress, constitutive activation of this defense pathway resulted in substantial developmental costs, including a 14–19% reduction in plant height, leaf shortening, and reduced grain numbers per spike. Importantly, the overaccumulation of jasmonate in the embryogenic calli inhibited somatic embryogenesis and reduced transformation efficiencies up to 25-fold (to 0.3% in bread wheat and 0.5% in emmer), emphasizing the critical need for tissue-specific or inducible promoters to harness the defensive properties of jasmonates without affecting crop production. The study highlights the importance of promoter selection and tissue-specific expression, a theme shared with other contributions exploring controlled gene expression strategies.

Alterations in traits such as plant architecture and developmental timing can be successfully engineered using tissue-specific promoters. Do et al. [10] developed a simple and cheap transformation protocol for elite Korean rice cultivars (‘Dongjin’ and ‘Samkwang’) using seed-derived calli and a liquid cocultivation medium (N6CI) that does not require expensive centrifugation. They constructed an antisense construct of the apple *FLOWERING LOCUS T-like* (*MdFT1*) gene under the control of the sugar-inducible rice α-amylase promoter, Ramy3D, with high transformation efficiencies (53.4% and 47.4%). The stable antisense expression of this apple florigen ortholog delayed heading date (67.1 d vs. 65.6 d) and modulated plant height, starch qualities, and free amino acid levels in the transgenic grain, suggesting that woody perennial genes can be functionally integrated to optimize cereal architectures. The study demonstrates how inducible and tissue-specific promoters mitigate pleiotropic penalties, complementing the regulatory insights presented later in this Special Issue.

To bypass transgenic regulations and public concerns altogether, transient synthetic biology is a highly promising frontier. Erokhina et al. [11] comprehensively reviewed the agricultural and biotechnological applications of artificial microRNAs (amiRNAs) and microRNA-encoded peptides (miPEPs). Whereas amiRNAs can be engineered to target and silence specific pathogen transcripts with extreme precision, the exogenous application of synthetic miPEPs represents a revolutionary, nontransgenic therapy. Spraying or irrigating plants with designed miPEPs specifically stimulates the transcription of their cognate pri-miRNAs (e.g., miPEP166g for root biomass, miPEP172c for nodulation, or miPEP172b for cold tolerance) without integrating foreign DNA into the host genome, offering a public-friendly and sustainable tool for precision crop management. This nontransgenic strategy aligns with global trends toward public-friendly biotechnologies and complements the genome-editing regulatory analysis included in this Special Issue.

Ultimately, the successful translation of these scientific breakthroughs into commercial crop production worldwide will heavily depend on the functionality and flexibility of regulatory systems. Sánchez [12] provides a critical global perspective on the market entry of genome-edited plants, emphasizing the essential function of Chile as a facilitator. Sánchez contrasted restrictive, process-based GMO regulations with flexible, science-driven, product-based frameworks that exempt edited crops from GMO oversight if they contain no foreign DNA insertions. Chile has pioneered an agile, case-by-case consultation system that has evaluated dozens of submissions utilizing advanced multiplex genome editing (analyzing 57 applications representing 1103 lines, of which 51% utilized multiplexing). Chile’s flexible regulatory environment, and given its position as a leading counter-season seed exporter, has accelerated the testing, registration, and commercial inclusion of NGT crops globally. This regulatory flexibility forms a crucial bridge between laboratory innovation and commercial deployment, completing the continuum of technological advances showcased in this Special Issue.

## 2. Conclusions

The studies compiled in this Special Issue establish a continuum of innovation, demonstrating that resolving bottlenecks in basic cellular physiology and propagation efficiency is a prerequisite for the successful deployment of CRISPR/Cas9 editing and advanced crop biotechnology. By connecting cellular microenvironments, translational boosters, single-cell editing platforms, and science-driven regulatory pathways, these twelve papers lay a highly cohesive and robust foundation for securing the global food supply in the future.
